# Sustaining collegiate recovery programs: a literature review and SWOT analysis

**DOI:** 10.3389/fpubh.2026.1898555

**Published:** 2026-09-03

**Authors:** Michelle K. Strong, Lauren E. Lewis

**Affiliations:** Department of Community, Family, and Addiction Sciences, Texas Tech University, Lubbock, TX, United States

**Keywords:** addiction recovery, behavioral addiction, collegiate recovery, higher education, organizational sustainability, substance use disorders, SWOT analysis

## Abstract

Substance use disorder and behavioral addictions remain significant concerns within higher education, yet little research has examined Collegiate Recovery Programs (CRPs) from an organizational perspective. This narrative literature review synthesized 71 peer reviewed studies to examine the internal and external organizational factors influencing the long term sustainability of CRPs using a Strengths, Weaknesses, Opportunities, and Threats (SWOT) framework. Following a comprehensive literature search, findings were analyzed using deductive thematic synthesis to identify recurring organizational characteristics within each SWOT domain. Five organizational strengths, five weaknesses, several external opportunities, and six external threats emerged across the body of literature. Organizational strengths included recovery supportive cultures, peer driven infrastructures, integration of recovery with student success initiatives, sustained recovery support, and institutional value. Recurring weaknesses included financial instability, organizational heterogeneity, inconsistent institutional integration, staffing variability, and limited organizational evaluation. External opportunities centered on expansion into diverse educational settings, institutional partnerships, workforce development, and strengthening the evidence base through longitudinal and financial evaluation, whereas threats included recovery stigma, unstable funding environments, inconsistent policy support, limited stakeholder awareness, and changing higher education environments. Collectively, the findings suggest that the sustainability of CRPs depends not only on positive student outcomes but also on organizational capacity, institutional integration, strategic partnerships, and measurable organizational performance. This review advances an organizational framework for understanding the broad impact and long term sustainability of CRPs and provides a foundation for future research, policy, and practice.

## Sustaining collegiate recovery programs: a literature review and SWOT analysis

Substance use disorder (SUD) and other behavioral addictions such as gambling and eating disorders are significant concerns on university campuses in the United States with approximately 21% of college students meeting DSM-5 criteria for a SUD (1), an estimated 5% experiencing problem gambling behaviors (2), and more than 7% reporting a recent eating disorder diagnosis or treatment (3). Additionally, misuse of prescription medications, cannabis, and various other substances have increased over the past decade, following changes in cannabis legalization and increased acceptability in social norms surrounding substance use (4). For students in recovery from SUD or other behavioral addictions, the college campus increases risk of relapse due to easy access to substances, social acceptance of behavioral addictions, and limited visibility of recovery resources (5).

Collegiate recovery programs (CRPs) emerged in response to the growing recognition that traditional higher education environments present significant risks for students in recovery from SUD and behavioral addictions. The earliest formal CRPs were established in the late 1970s and 1980s at Brown University, Rutgers University, and Texas Tech University, each developing independently yet grounded in similar principles of peer support, accountability, and academic success (6, 7). Early CRPs emphasized abstinence based recovery, participation in mutual aid groups, and the creation of substance free social environments, positioning recovery not as a barrier to higher education but as a protective factor and a supported identity on campus (8). Although abstinence based recovery continues to represent the predominant model within many CRPs, the field has evolved to recognize multiple pathways to recovery, with some programs incorporating harm reduction philosophies, moderation approaches, medication supported recovery, and other individualized recovery pathways that reflect the diverse needs and goals of college students (8, 9). This evolution has broadened the conceptualization of recovery within higher education while maintaining the central emphasis on student wellbeing, academic success, and recovery supportive communities.

Over the past two decades, CRPs have expanded rapidly across institutions of higher education in the United States (10). From fewer than 10 identified programs in the early 2000s, the number of CRPs has grown to more than 175 campuses nationwide, with additional programs emerging in Canada and internationally (11). This growth has been driven by several factors, including increased college enrollment among students in recovery, improved recognition of recovery as a long term process rather than an acute treatment outcome, and accumulating evidence linking participation in CRPs to positive academic and recovery related outcomes (9).

Collegiate recovery programs are most effectively understood not simply as on campus student support services, but as critical organizational systems within institutions of higher education that contribute to public health and workforce development. Currently, CRPs operate at the intersection of academic affairs, student affairs, and community based recovery systems, requiring coordination across multiple organizations and funding streams. Much like a non-profit organization, CRPs impact and sustainability are shaped not only by individual outcomes, such as academic success, sustained recovery, and career readiness, but also by organizational structures, institutional leadership commitment, resource allocation, and program culture (12). Viewing CRPs through an organizational lens enables a comprehensive analysis of how internal factors and external environmental factors shape long term sustainability, as well as their strategic alignment with institutional priorities that advance holistic student success, workforce development, and public health outcomes (13). Without an organizational analysis, evaluations of CRPs risk overemphasizing individual outcomes and societal impact without considering their ability to sustain themselves.

A Strengths, Weaknesses, Opportunities, and Threats (SWOT) analysis offers a systematic and well established approach for examining organizations within their internal and external environments (14). Widely used in strategic planning across business and adopted by higher education and public health settings, a SWOT analysis provides an exceptional tool to examine the existing body of collegiate recovery literature and identify internal strengths and weaknesses and external opportunities and threats addressing the sustainability of the programs (15). In higher education research, SWOT analyses have been used to assess student services, academic programs, and institutional initiatives in times of uncertainty (16). Within public health, SWOT analyses have been applied to evaluate community based interventions and recovery oriented systems of care, offering insight into how organizations interact with sociopolitical environments to shape outcomes (16). Therefore, applying a SWOT analysis provides a comprehensive strategic framework for identifying the organizational factors that promote or hinder the sustainability of CRPs operating as non-profit organizations within institutions of higher education.

Guided by organizational strategy perspectives, this review addresses the overarching research question: What are the internal strengths, internal weaknesses, external opportunities, and external threats influencing the organizational sustainability of CRPs? To address this question, four sub-questions were developed to align with the four quadrants of the SWOT analysis framework. First, what are the internal organizational strengths that enhance the sustainability of CRPs? Second, what internal organizational weaknesses limit the long term sustainability of CRPs? Third, what external opportunities that have the potential to enhance the sustainability of CRPs? And lastly, what external threats jeopardize the organizational sustainability of CRPs?

By structuring the review around these four research questions, this paper provides an assessment of CRPs that move beyond descriptive program summaries and individual student outcomes. This approach identifies actionable insights for institutional leaders, researchers, and policymakers while contributing to the body of collegiate recovery and recovery science literature.

## Methods

This paper utilized a narrative literature review design to examine CRPs through an organizational lens to identify factors for each SWOT quadrant. Narrative literature reviews are well suited to synthesizing diverse bodies of literature to develop conceptual understanding and generate new perspectives (17). To enhance methodological rigor and transparency, a structured SWOT analytical framework was incorporated to systematically organize findings according to predefined organizational categories (18). Following completion of a thematic synthesis, the findings within each SWOT quadrant were synthesized to identify recurring organizational themes across the literature to develop an understanding of the factors influencing the long term sustainability of CRPs.

### Literature search

A comprehensive literature search was conducted in December 2025 to identify peer reviewed studies examining CRPs. Searches were conducted across multiple interdisciplinary databases including Google Scholar, PsycINFO, PubMed, Scopus, ERIC, and Academic Search Complete. The use of multiple databases was intended to maximize results with a cross disciplinary focus. The search term strings are presented in Table 1. Consistent with the purpose of a narrative literature review, the search was limited to peer reviewed publications to ensure that all included studies had undergone scholarly evaluation and met established standards of methodological rigor (17). Eligible studies included empirical and conceptual articles published in peer reviewed journals that discussed CRPs at colleges and universities in the United States and Canada. Including both empirical and conceptual literature allowed for a comprehensive examination of organizational characteristics relevant to the strengths, weaknesses, opportunities, and threats associated with CRPs. Studies conducted in non-collegiate recovery settings, such as community recovery organizations or recovery high schools, were excluded because they operate within substantially different organizational contexts. Gray literature, dissertations, conference proceedings, and other publications that were not peer reviewed were excluded to maintain methodological consistency and ensure that the synthesis was based on rigorously reviewed scholarly evidence. There were no timeline parameters on the search given the recent emergence of the field of collegiate recovery.

**Table 1:** Database search terms.

“Collegiate recovery”; “collegiate” and “recovery”; “college” and “recovery” and “addiction”; “collegiate recovery programs”; “university” and “recovery” and “addiction”; “students” and “college” and “recovery”; “collegiate recovery” and “program”; “collegiate” and “recovery” and “program”; “recovery” and “college”

The article selection and review process followed a multistage screening procedure. Initial database searches yielded 281 articles, which were screened at the title and abstract level for relevance to collegiate recovery using Rayyan literature review software. Eighty-five articles underwent full text review to assess alignment with inclusion parameters. Following this process, a total of 71 peer reviewed articles were retained for synthesis and coding (Appendix A). Each included article was reviewed in detail and coded for relevance to the four quadrants of the organizational SWOT analysis. This detailed screening and review process ensured that the literature review reflected the breadth of the collegiate recovery literature while remaining aligned with the organizational SWOT focus.

### Thematic synthesis and SWOT analysis

Following study selection, the full text of each included article was reviewed in its entirety. Relevant findings pertaining to internal and external organizational characteristics were manually extracted for analysis. Because the objective of this narrative review was to synthesize organizational characteristics across a heterogeneous body of literature, a thematic synthesis approach was employed to identify patterns and relationships among findings (17, 19).

Analysis was conducted using a deductive thematic synthesis guided by the SWOT framework as the *a priori* analytical structure (20). Prior to coding, four predefined analytical domains corresponding to the SWOT quadrants were established. Strengths were operationalized as internal organizational factors that enhance the long term sustainability of CRPs, whereas weaknesses represented internal organizational factors that limit organizational sustainability. Opportunities were defined as external opportunities to strengthen the sustainability of CRPs. Threats represented external environmental conditions that jeopardize sustainability.

Within each domain, findings were compared using an iterative process of constant comparison to identify consistent themes across studies (19). Consistent with thematic synthesis, coding emphasized the conceptual significance of findings rather than their frequency of occurrence, allowing organizational characteristics of importance to be retained regardless of the number of studies in which they appeared (19). This analytical approach facilitated the development of higher order themes while preserving the organizational structure underlying the SWOT framework (16, 19). The higher order organizational themes identified through thematic synthesis are summarized in Table 2.

**Table 2:** Summary of themes across SWOT domains.

SWOT domain	Higher order theme	Description of theme
Strengths	Recovery supportive culture	Program culture on campus providing peer support, accountability, belonging, substance free social events and multiple paths to recovery
	Peer driven organizational infrastructure	Peer mentorship, mutual aid groups, recovery capital
	Organizational integration of recovery and student success	Recovery embedding in the academic mission contributing to GPA, persistence, retention, graduation, and student engagement
	Organizational capacity to sustain recovery	Recovery supportive norms, program expectations, recovery identity, reduced relapse risk, recovery capital
	Institutional value	Contribution to the university through tuition retention, reduced institutional costs, public health, and workforce development
Weaknesses	Financial instability and funding dependence	Reliance on grants, donations, temporary funding
	Organizational heterogeneity and lack of standardization	Wide variation in program models, governance, staffing, programming, and membership limiting evaluation and comparison among programs
	Inconsistent institutional integration	Variable administrative placement, organizational visibility, reporting structures, and cross campus collaboration
	Staffing variability and lack of professionalization	Variability from student/volunteer staff to recovery professionals, lack of standardized leadership competencies, professional development pathways, or formal guidance
	Limited organizational evaluation and longitudinal outcomes	Limited evidence of post graduation and longitudinal data measuring organizational effectiveness
Opportunities	Expansion into community colleges and 2-year institutions	Increasing recognition of recovery support across diverse educational settings
	Expanded institutional and community partnerships	Greater collaboration among all campus offices, community organizations, and external stakeholders
	Recovery education awareness	Faculty and staff education, stigma reduction, campus awareness, increased visibility
	Workforce development and career readiness	Emerging emphasis on career readiness and the college to work transition and organizational assessment to demonstrate value
	Financial and organizational evaluation	Need for ROI analysis, financial metrics
	Longitudinal research and post graduation outcomes	Need for long term evaluation of recovery, placement, career trajectories, and wellbeing
Threats	Recovery stigma and substance normalizing campus cultures	Campus environments that normalize addictive behaviors while limiting recovery visibility
	Inconsistent policy support	Lack of federal or state policies supporting student recovery affecting institutional policy
	Unstable external funding environments	Dependence on changing grants priorities, philanthropic interests, and economic conditions
	Limited stakeholder awareness	Limited understanding among university leaders, employers, policymakers, community partners, and donors
	Organizational growth and standardization challenges	Lack of common standards, operational consistency, and evaluation as the field expands
	Evolving higher education environment	Financial pressures, changing enrollment, shifting priorities, demographics, and competition for limited institutional resources

**Figure 1:**
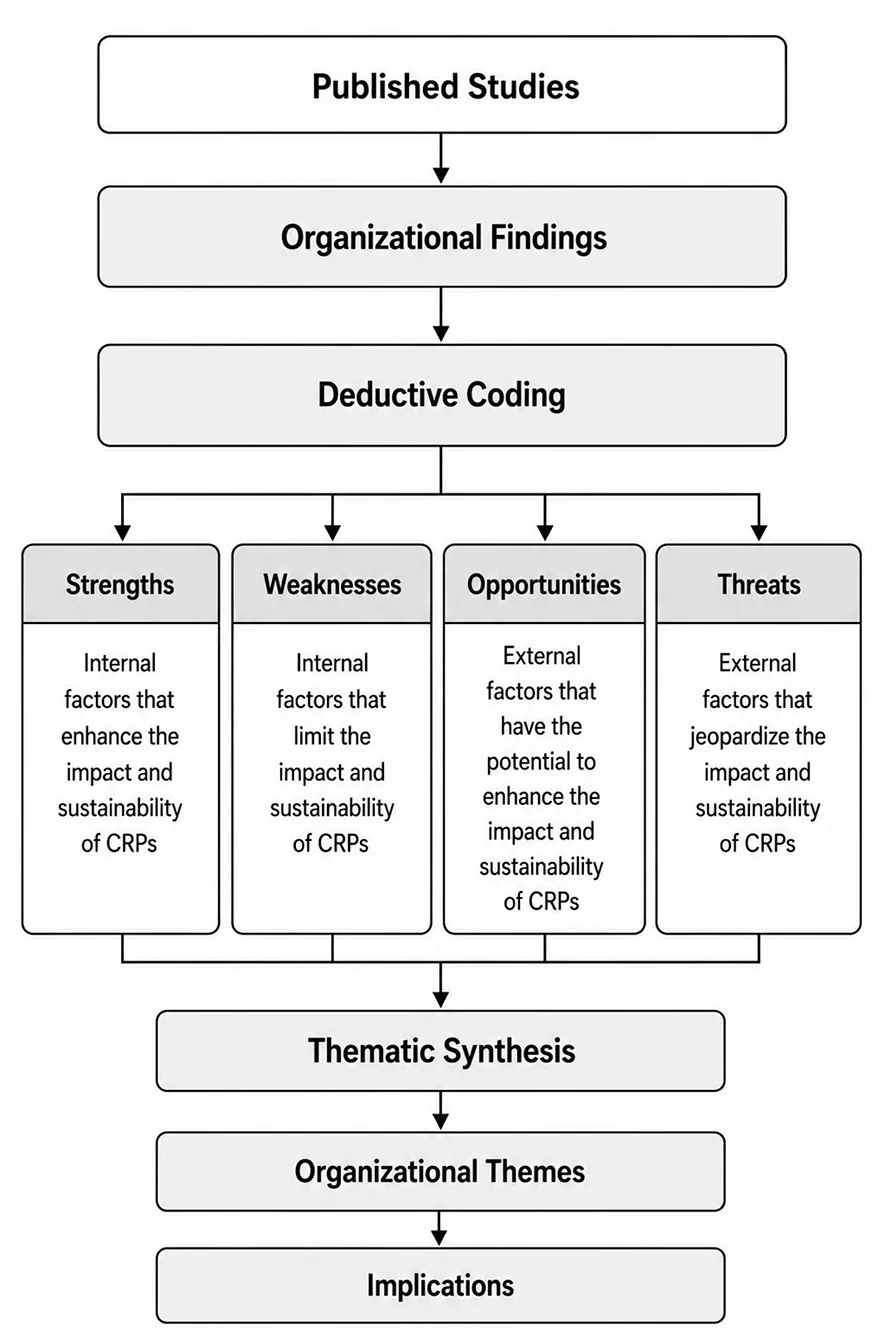
Thematic synthesis and SWOT theme development.

The coding and thematic synthesis were conducted by the first author as part of a graduate portfolio research requirement. To enhance analytic rigor, the coding process was iterative, with repeated review of the original articles throughout analysis to verify code assignment, refine thematic interpretation, and ensure that synthesized findings accurately reflected the literature. The evolving coding framework, thematic development, and assignment of findings to the SWOT quadrants were reviewed through regular consultation with the second author, who served as faculty advisor and provided methodological oversight. Rather than independently recoding the studies, the second author critically reviewed the coding framework, thematic organization, and conceptual alignment of findings within the SWOT structure to enhance the credibility and trustworthiness of the analysis. Following completion of coding, themes within each SWOT quadrant were reviewed to develop an integrated understanding of the internal and external organizational factors influencing the impact and sustainability of CRPs (Figure 1).

## Findings

**Figure 2:**
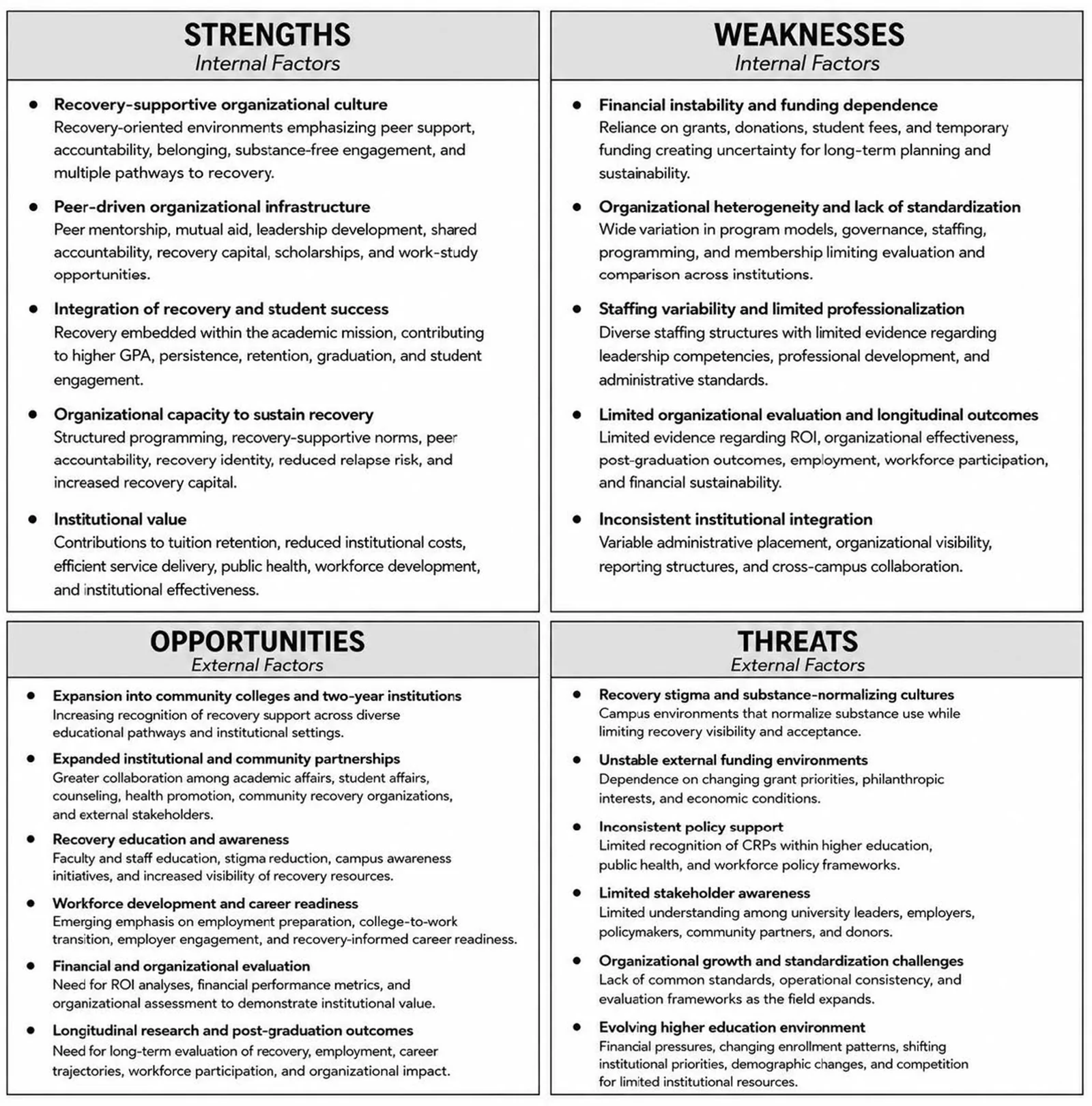
Collegiate recovery programs SWOT analysis.

The narrative synthesis of 71 peer reviewed studies identified a series of recurring organizational characteristics that were categorized within the four quadrants of the SWOT framework. Across the literature, findings consistently clustered into higher order themes representing the internal strengths and weaknesses of CRPs, as well as the external opportunities and threats influencing their long term sustainability. Because the findings represent the results of a thematic synthesis conducted across the included studies, they are presented as synthesized organizational themes within the SWOT quadrants rather than sequential summaries of articles (Figure 2).

### Strengths

Synthesis of the literature identified five higher order organizational strengths that contribute to the long term sustainability of CRPs; recovery supportive organizational culture; peer driven organizational infrastructure; integration of recovery and student success; organizational capacity to sustain recovery, and institutional value. Together, these strengths reflect organizational characteristics that support long term organizational sustainability while simultaneously supporting student recovery and advancing institutional priorities of holistic student success and workforce development.

Recovery supportive organizational culture emerged as the most consistently reported organizational strength across the literature. Collegiate recovery programs intentionally cultivate campus environments in which recovery is visible, socially supported, and integrated into the collegiate experience through peer support, accountability, and substance free social engagement. Unlike broader campus cultures that often normalize substance use and other behavioral addictions, CRPs establish recovery oriented communities that foster belonging, strengthen recovery identity, reduce social isolation, and encourage continued engagement in recovery. These organizational cultures also reinforce accountability through expectations related to academic performance, recovery participation, leadership development, and community involvement while increasingly recognizing multiple pathways to recovery. The body of literature supports a broad theme identifying recovery supportive culture as the organizational foundation upon which successful CRPs are built.

Peer driven organizational infrastructure also emerged strongly as a defining strength of CRPs. Rather than relying exclusively on professional or clinical service delivery, the literature consistently reports peer mentorship, mutual aid meetings, shared accountability, and recovery community norms as central organizational components. These peer based structures were associated with increased student engagement, leadership development, and community ownership while facilitating the development of recovery capital through access to personal, social, and community resources that support sustained recovery. A few studies further described tangible organizational support, including scholarships and work study opportunities, as additional mechanisms that strengthen students' recovery capital.

The integration of recovery support within the academic mission of higher education represented another recurring organizational strength. Rather than positioning recovery separately from educational achievement, CRPs embedded recovery support within students' academic experiences and campus engagement. Across multiple studies, students participating in CRPs consistently demonstrated strong academic outcomes, including high grade point averages, academic persistence, retention, and graduation rates. Together, these findings associate academic success with organizational structures that intentionally integrate peer accountability, recovery oriented programming, and academic support into the collegiate experience.

Further identified as strong organizational characteristics that promote sustained recovery within non-clinical educational settings was low relapse rates. Although relapse prevention was not described as the primary organizational mission of CRPs, studies consistently reported that peer accountability, recovery supportive norms, structured programming, and mutual support networks created environments that reinforced long term recovery behaviors. Participation in CRPs was repeatedly associated with reduced relapse risk, strengthened recovery identity, and increased recovery capital among students recovering from SUD and behavioral addictions. These findings demonstrate that organizational environments emphasizing connection, accountability, and recovery engagement consistently support students as they pursue both academic and personal goals.

The literature also identified organizational strengths that contributed to their university as a whole. Higher retention and graduation rates were associated with increased tuition retention and reduced institutional costs related to student attrition. Additional organizational benefits included reduced utilization of crisis oriented campus services, disciplinary systems, and emergency interventions. Several studies further characterized peer driven organizational structures as efficient approaches to delivering recovery support while reducing reliance on traditional student support services. These findings show the organizational strength of the CRPs as contributors to institutional value through their alignment with student success initiatives, public health priorities, and workforce development objectives.

### Weaknesses

Five themes emerged as organizational weaknesses that constrain the sustainability of CRPs; financial instability and funding dependence; organizational heterogeneity and lack of standardization; staffing variability and limited professionalization; limited organizational evaluation and longitudinal outcomes, and inconsistent institutional integration. These weaknesses reflect internal characteristics that may reduce organizational capacity and hinder long term sustainability.

Financial instability emerged as the most consistently reported organizational weakness across collegiate recovery literature. Multiple studies described CRPs as relying on grants, philanthropic donations, student fees, or short term institutional allocations rather than recurring line item funding within university. Dependence on temporary or externally generated funding creates uncertainty regarding long term planning, staffing continuity, program expansion, and the future of the programs. Financial uncertainty was also associated with increased organizational vulnerability during periods of institutional restructuring, shifting funding priorities, and broader economic instability.

Organizational heterogeneity likewise emerged as a recurring weakness. Although CRPs share common recovery oriented principles, studies consistently described considerable variation in membership requirements, programming, staffing, governance, administrative structures, and service delivery. This variability limited opportunities to compare programs systematically, establish standardized organizational performance measures, evaluate ROI, and identify evidence informed organizational practices. Collectively, the literature identifies the absence of consistent organizational models as a continuing challenge for evaluating and advancing the stability of CRPs.

Variation in staffing models and program leadership represented a third organizational weakness. Programs ranged from student led and volunteer operated initiatives to fully staffed recovery centers employing dedicated recovery professionals. Despite this diversity, relatively little evidence addressed standardized leadership competencies, administrative structures, professional development pathways, or formal guidance for CRP administration. As a result, staffing variability and limited professionalization emerged as recurring organizational characteristics that directly influence the lack of organizational consistency and operational sustainability.

The literature also revealed important limitations in organizational evaluation and longitudinal assessment. Although numerous studies documented positive academic and recovery related outcomes during students' participation in CRPs, there was little evidence that examined post graduation recovery, employment outcomes, workforce participation, organizational performance, or financial sustainability. Similarly, evidence describing organizational effectiveness, institutional value, ROI, and long term financial sustainability remained limited. Without organizational evaluation, the long term organizational sustainability of CRPs remains incompletely understood.

A final organizational weakness involved inconsistent institutional integration. Across the reviewed literature, CRPs operated within a wide variety of administrative structures, including counseling centers, student affairs divisions, health promotion offices, and stand alone programs. Differences in institutional placement were associated with variation in organizational visibility, reporting relationships, access to institutional resources, and opportunities for cross campus collaboration. Together, these findings indicate that institutional integration remains highly variable across colleges and universities and continues to represent an important organizational challenge within the collegiate recovery landscape directly impacting long terms sustainability of the programs.

### Opportunities

Several recurring external developments and future directions emerged from the literature that present opportunities for creating sustainability of CRPs. Across the reviewed studies, authors identified expansion of collegiate recovery into community colleges and other 2 year educational settings, expanded campus and community partnerships, increased institutional integration, the need for career readiness, recovery education and awareness, and systems level organizational development as important areas for continued growth. The literature also identified limited financial and longitudinal outcome data as significant gaps, highlighting opportunities for future research to strengthen the evidence base supporting CRPs.

Expansion of collegiate recovery beyond traditional 4 year universities emerged as an important theme. Several studies described the establishment of recovery support services within community colleges, technical colleges, and transfer pathways, reflecting growing recognition of the increased enrollment in community colleges and students in recovery. Further described was the development of campus collaborations designed to increase access to recovery resources and extend collegiate recovery services to broader student populations across university systems.

The literature also identified the lack of longitudinal data as an emerging area of emphasis. Although CRPs consistently reported positive academic and recovery outcomes, several authors noted comparatively little data being captured post graduation to measure long term success. This type of data presents a significant opportunity to demonstrate ROI to secure funding for continued sustainability.

Expansion of campus partnerships likewise emerged throughout the literature suggesting the opportunity of institutional collaboration among admissions, orientation, academic affairs, student affairs, counseling services, disability services, health promotion, and student wellness initiatives, reflecting broader institutional engagement with collegiate recovery. Also on campus, recovery education and awareness also emerged as recurring themes. Faculty and staff education, campus wide awareness initiatives, stigma reduction efforts, and increased visibility of recovery resources were frequently described as strategies for improving understanding of recovery and strengthening campus support for students in recovery. Across the literature, these initiatives reflected increasing recognition of recovery as an important component of student health, wellbeing, and campus inclusion, which can directly impact sustainability.

Alongside these developments, several authors identified the limited availability of financial data, organizational performance measures, and ROI analyses as important gaps in the current evidence base. Additionally, relatively few studies examined post graduation employment outcomes, or other longitudinal indicators of program effectiveness. Collectively, these findings identify financial evaluation and long term outcome research as important priorities for strengthening the evidence supporting CRP organizational sustainability.

### Threats

The literature identified several themes representing external conditions that hinder CRPs; recovery stigma and substance normalizing campus cultures; unstable external funding environments; inconsistent policy support; limited stakeholder awareness; challenges associated with organizational growth and standardization; and broader changes within higher education.

Recovery stigma associated with SUD and behavioral addiction normalizing campus cultures emerged as the most frequently reported external challenge. Multiple studies described collegiate environments in which the use of substances and other disordered behavior remain central components of student social life, increasing exposure to relapse risks for students in recovery. The literature also documented persistent misconceptions surrounding SUD and recovery from behavioral addictions that contributed to limited visibility of recovery resources and reduced understanding among students, faculty, administrators, and the broader campus community. Together, these findings indicate that stigma and campus norms surrounding addictive behaviors cause threats to the long term sustainability of CRPs.

The literature also identified uncertainty surrounding external funding as a recurring threat causing cuts or elimination of entire programs. Noted are funding sources being influenced by changing policies, economic conditions, and philanthropic interests. Researchers described uncertainty regarding the continuation of grant funded initiatives and other external financial support, reflecting variability in the broader funding environment.

Inconsistent policy recognition also emerged across the reviewed literature. Relatively few state or federal policies specifically identify CRPs within higher education, workforce development, or public health initiatives. Studies further described variation in institutional policies related to recovery support, student health, and behavioral health services, resulting in differing levels of institutional recognition and support across colleges and universities.

Limited awareness of collegiate recovery among external stakeholders was another recurring finding. Studies described limited understanding of CRPs among students, university leadership, employers, policymakers, community organizations, and potential donors. The literature also noted limited availability of organizational performance measures and longitudinal outcome data, which may influence external understanding of collegiate recovery and its role within higher education.

Also noted in the literature are challenges associated with program structure. Studies described considerable variation in organizational models, operational practices, and evaluation approaches across institutions, reflecting the absence of widely accepted program standards, membership, or common frameworks. These findings illustrate the diversity of organizational approaches currently represented within CRPs which can significantly impact long term sustainability.

Finally, the reviewed literature described broader changes within higher education that may influence the future development of CRPs. Shifting enrollment patterns, evolving institutional priorities, increasing financial pressures, changing student demographics, and competing demands for institutional resources were repeatedly identified as contextual conditions affecting colleges and universities. These findings demonstrate that CRPs continue to operate within a dynamic higher education environment with continued external threats of changing institutional, financial, and societal conditions.

## Discussion

To our knowledge, this review is the first to examine CRPs through an organizational strategy lens using a SWOT framework. While previous research has largely emphasized student level outcomes such as recovery maintenance, academic success, and wellbeing, the present synthesis extends the literature by identifying the internal organizational characteristics and external environmental conditions that collectively influence the sustainability of CRPs. The findings suggest that organizational sustainability is shaped not by any single factor, but by the interaction between internal organizational capacity and external systems environments.

A central finding of this review is that the organizational strengths identified across the literature position CRPs to capitalize on emerging opportunities within higher education, workforce development, and public health. Recovery supportive organizational cultures, peer-driven infrastructures, integration with institutional missions, and organizational capacity to sustain recovery provide strategic advantages that extend beyond supporting individual students. Together, these strengths align closely with institutional priorities related to holistic student success, public health, and workforce preparation. Rather than functioning solely as recovery support services, the findings suggest that CRPs operate as organizational assets capable of advancing institutional missions.

Equally important is the interaction between organizational weaknesses and external threats. The review consistently identified funding instability, organizational heterogeneity, inconsistent institutional integration, staffing variability, and limited longitudinal outcome data as internal weaknesses that increase organizational vulnerability. These weaknesses intersect with external threats, including stigma, shifting funding priorities, inconsistent policy support, and limited public awareness, creating conditions that may constrain organizational growth regardless of positive student outcomes. This suggests that demonstrating effectiveness at the student level alone is insufficient to ensure long term organizational sustainability. Instead, sustainability appears to depend upon a CRPs ability to demonstrate strategic value, institutional alignment, and measurable ROI to university leadership, policymakers, and external funding partners.

The SWOT framework further illustrates that organizational strengths and weaknesses do not operate independently of opportunities and threats but rather influence a CRPs capacity to respond to its external environment. Programs possessing strong recovery supportive cultures, effective peer infrastructures, and institutional integration appear better positioned to leverage opportunities such as workforce development partnerships, expansion into community colleges, recovery informed career readiness initiatives, and system level institutional adoption. Conversely, organizations lacking stable funding, standardized evaluation metrics, or institutional integration may experience greater difficulty capitalizing on these same opportunities. Viewing these relationships through a strategic organizational framework provides a more comprehensive understanding of CRPs than evaluations focused exclusively on individual student outcomes.

The most significant contribution of this review is the reframing of CRPs as organizations rather than auxiliary student services. Organizational strategy literature emphasizes that long term organizational success depends upon the alignment of internal capabilities with external environmental demands (21, 22). Applying this perspective to collegiate recovery suggests that the future success of CRPs will depend not only on their continued ability to improve student outcomes, but also on their ability to institutionalize the programs within higher education systems, demonstrate organizational performance, diversify funding streams, establish sustainable partnerships, and communicate their value to institutional leaders, employers, policymakers, and community stakeholders. As a result, organizational sustainability should be considered an essential outcome alongside student recovery and academic success in future collegiate recovery research.

### Toward an organizational framework for collegiate recovery

The findings of this review suggest that CRPs are best understood through a broader organizational framework in which student outcomes and organizational sustainability are interconnected rather than independent constructs. Positive student outcomes, including sustained recovery, academic achievement, persistence, graduation, and recovery capital development, represent the most visible indicators of program effectiveness. However, these outcomes also serve as evidence of organizational effectiveness by demonstrating that CRPs successfully fulfill their intended mission. As organizations consistently produce positive outcomes, they are better positioned to integrate within institutional strategic priorities related to student success, public health, and workforce development. Increased institutional integration, in turn, strengthens organizational legitimacy by reinforcing the value of CRPs to university leaders, policymakers, funding agencies, employers, and other stakeholders. Organizational legitimacy enhances an institution's capacity to secure stable financial resources, establish strategic partnerships, diversify funding streams, and attract philanthropic investment. These organizational processes contribute to long term sustainability by enabling CRPs to expand their reach, strengthen organizational capacity, and remain responsive to evolving institutional and societal needs. Viewed from this perspective, student outcomes should not be considered the endpoint of collegiate recovery but rather the foundation upon which organizational effectiveness, institutional legitimacy, and long term sustainability are built.

### Implications for higher education

The findings suggest that institutions of higher education should begin viewing CRPs as strategic organizational assets rather than ancillary student support services. Across the literature, the organizational strengths identified in this review, including recovery supportive organizational cultures, peer driven infrastructures, and integration of recovery with student success initiatives, align closely with institutional priorities related to student success and wellbeing. Positioning CRPs within broader institutional strategic plans would therefore strengthen organizational legitimacy while reinforcing their contribution to institutional performance metrics that increasingly influence accreditation, funding, and public accountability.

The findings further suggest that enhanced sustainability would come upon integrating CRPs into the core organizational infrastructure of universities rather than maintaining them as peripheral or discretionary services. Stable administrative placement, dedicated budget allocations, organizational assessment systems, and cross campus collaboration emerged as important opportunities that may enhance long term sustainability. Embedding CRPs within strategic planning processes also creates opportunities to strengthen partnerships among academic affairs, student affairs, counseling services, health promotion, disability services, and career development offices. Such integration positions recovery as a shared institutional responsibility rather than an ancillary function.

### Implications for public health and workforce development

Viewing CRPs through an organizational strategy lens also expands their relevance beyond higher education into public health and workforce development. Rather than functioning solely as campus recovery initiatives, CRPs operate as upstream public health interventions that support sustained recovery during a critical developmental period while simultaneously increasing educational attainment and workforce participation. These intersecting outcomes position CRPs to contribute to broader public health and economic development initiatives.

The findings further identify career readiness as one of the most significant opportunities currently underrepresented within the collegiate recovery literature. Although educational attainment has long been recognized as a determinant of sustained recovery, relatively little attention has been devoted to preparing students for successful transitions into employment. Integrating recovery informed career readiness, employer engagement, leadership development, and college-to-work transition planning into CRPs represents a logical extension of existing organizational strengths. Such initiatives may strengthen employment outcomes while simultaneously increasing organizational value through stronger alignment with institutional workforce priorities.

Employer partnerships similarly emerged as an important organizational opportunity. Developing relationships with recovery informed employers, industry partners, alumni, and philanthropic organizations may diversify funding streams while expanding employment opportunities for graduates. These partnerships also create opportunities for higher education institutions to promote recovery friendly workplaces, strengthen regional workforce pipelines, and demonstrate broader societal impact beyond traditional academic metrics.

### Implications for research

This review highlights several priorities for future collegiate recovery research that extend beyond documenting student level outcomes. Most notably, there remains a critical need for longitudinal research examining career readiness along with employment placement data, as well as measuring post graduation career trajectories and overall wellbeing measures. Without this data, CRPs remain limited in their ability to demonstrate the long term institutional, economic, and societal impact associated with CRPs.

The findings also underscore the importance of investigating organizational characteristics as independent variables influencing program effectiveness. Considerable variability exists among CRPs with respect to staffing models, leadership structures, administrative placement, funding mechanisms, recovery philosophies, institutional size, and organizational maturity. Future comparative research should examine how these organizational variables influence student outcomes, organizational sustainability, institutional integration, and scalability. Such work would substantially strengthen the application of organizational strategy within collegiate recovery research.

Another important implication concerns the development of standardized organizational metrics. The SWOT analysis identified limited evidence regarding organizational performance indicators, financial sustainability, strategic planning, and CRP ROI. Future research should prioritize the development and validation of organizational assessment frameworks capable of evaluating not only student outcomes but also organizational capacity, institutional value, funding diversification, stakeholder engagement, and long term sustainability. Establishing common organizational metrics would strengthen evidence based decision making while facilitating comparisons across institutions.

Finally, the heterogeneity of CRPs highlights the need for interdisciplinary scholarship integrating higher education, addiction and recovery science, non-profit management, implementation science, public health, organizational behavior, and workforce development. Such collaborations may provide a more comprehensive understanding of how organizational systems influence both student recovery outcomes and CRP performance.

### Implications for policy

The SWOT analysis suggests that current policy frameworks have not kept pace with the organizational evolution of CRPs. Although CRPs consistently demonstrate positive academic and recovery related outcomes, their continued reliance on discretionary funding and fragmented institutional support creates organizational vulnerability that limits long term sustainability. Policy initiatives should therefore shift beyond supporting individual recovery services toward institutionalizing recovery as an essential component of higher education.

At the institutional level, policy should encourage the inclusion of CRPs within strategic funding models linked to holistic student success and workforce development. State and federal accountability systems increasingly reward institutions demonstrating measurable educational outcomes, creating opportunities to recognize CRPs as evidence informed contributors to institutional performance. Integrating organizational performance indicators into accountability frameworks may strengthen both institutional investment and long term sustainability.

Public health policy similarly presents opportunities to expand the organizational role of CRPs. Recovery is increasingly recognized as a community based process influenced by educational opportunity, supportive environments, and employment opportunity. Recognizing CRPs within public health planning and funding mechanisms would strengthen continuity of care while supporting broader objectives related to recovery capital, economic participation, and community wellbeing.

Finally, workforce policy represents an emerging area for organizational development. Incentivizing partnerships among higher education institutions, employers, workforce development agencies, and recovery organizations may strengthen employment pathways for students in recovery while promoting recovery friendly workplace practices. Such policies would extend the influence of CRPs beyond higher education and position them as contributors to regional economic development, workforce resilience, and population health.

## Limitations

Several limitations should be considered when analyzing the findings of this review. First, the use of a narrative literature review design, while well suited to synthesizing a heterogeneous and emerging body of literature, can limit replicability due to the positionality of the researcher. Unlike systematic reviews or meta-analyses, narrative reviews rely on interpretive coding and synthesis, which may introduce subjectivity despite the use of a structured SWOT framework. Although this study employed transparent inclusion criteria and methodology, the findings should be understood as informative rather than definitive.

Second is the restriction to peer reviewed published literature. Although limiting inclusion to peer reviewed studies strengthened the methodological rigor and quality of the evidence synthesized, this approach may have increased the potential for publication bias. Future reviews could incorporate gray literature to provide a more comprehensive understanding of the organizational characteristics related to impact and sustainability of all collegiate recovery literature.

Third, the extensive heterogeneity of CRPs models presents a significant limitation. The curricula and support offered vary widely in size, membership model, staffing, funding, and institutional placement, reflecting differences in campus culture and resources. This variability complicates program comparison and limits the ability to generalize findings across institutions. While such heterogeneity reiterates the need for organizational analysis, it also constrains efforts to identify the SWOT characteristics that are applicable to all CRPs.

Finally, a significant limitation of this study is the relative scarcity and inconsistency of evidence related to funding structures and long term financial sustainability. Although the literature presented cost benefit analyses, there was no evidence of budget size, revenue diversification, or the relationship between funding mechanisms and program outcomes. Much of the available evidence relies on self-reported data from collegiate recovery directors, descriptive case studies, or cross-sectional surveys, limiting the ability to draw causal inferences about how specific funding models influence program sustainability over time. As a result, funding is discussed in the literature as a program constraint rather than a core organizational variable. This gap is particularly consequential within the SWOT framework, where funding operates simultaneously as an internal weakness, an external threat, and a critical determinant of program sustainability. Without detailed financial data, it becomes difficult to assess the sustainability of CRPs, compare institutional commitment, or identify sustainable pathways for growth.

## Conclusion

This review extends the collegiate recovery literature by shifting the focus from outcomes to the organizational characteristics that influence the long term sustainability of CRPs. By applying a SWOT framework to 71 peer reviewed studies, this review identified the internal organizational strengths and weaknesses, as well as the external opportunities and threats, that collectively shape the strategic positioning of CRPs within higher education. In doing so, it provides one of the first comprehensive organizational analyses of CRPs, demonstrating that the sustainability of these programs depends not only on the societal impact of improving recovery and academic outcomes, but also on their capacity to establish ROI, secure sustainable resources, and adapt to an evolving higher education landscape.

The findings indicate that CRPs possess substantial organizational strengths, including recovery supportive cultures, peer driven infrastructures, integration with institutional student success priorities, and the capacity to promote sustained recovery within non-clinical educational settings. At the same time, the review identified organizational vulnerabilities related to funding instability, inconsistent institutional integration, heterogeneous program models, and limited longitudinal evaluation. Importantly, the SWOT analysis illustrates that these internal organizational characteristics influence a program's ability to capitalize on emerging opportunities, including workforce development, community college expansion, employer partnerships, recovery informed career readiness, and broader public health initiatives, while simultaneously shaping vulnerability to external threats such as stigma, policy instability, and shifting funding priorities.

In sum, these findings suggest that organizational sustainability should be viewed as an essential outcome of CRPs rather than merely a condition supporting student success. Positive recovery and academic outcomes establish organizational effectiveness, organizational effectiveness strengthens institutional integration and legitimacy, and institutional legitimacy enhances a CRP's capacity to secure resources, develop strategic partnerships, and sustain long term growth. Viewing CRPs through this organizational framework provides a more comprehensive understanding of how recovery supportive systems create value for students, higher education, and society while offering a conceptual foundation for future organizational research.

As institutions of higher education increasingly emphasize holistic student success and workforce development, CRPs are uniquely positioned to contribute to each of these priorities. Realizing this potential, however, will require moving beyond viewing CRPs as ancillary student support services toward recognizing them as strategic organizational assets that strengthen institutional performance while improving the lives of students in recovery. Future research should continue to investigate the organizational mechanisms that sustain these programs, develop standardized measures of organizational effectiveness, and evaluate long term institutional and societal outcomes. By advancing an organizational perspective of collegiate recovery, this review provides a foundation for the next generation of research, policy, and practice aimed at ensuring that recovery is not only supported on college campuses, but institutionalized as an enduring component of higher education, workforce development, and public health.

## Appendix A

**Table A1:** Literature reviewed.

References	Primary focus
Ashford et al. (23)	CRP – Integrated health model
Ashford et al. (24)	CRP – Meta-synthesis
Ashford et al. (25)	CRP – Eating disorders
Bannard et al. (26)	CRP – Directions for future research
Beeson et al. (27)	CRP – Social Cognitive Theory in social ecological framework
Bell et al. (28)	CRP – Current estimates of resources
Bell et al. (29)	CRP – Feedback on impact on students
Bell et al. (30)	CRP – Recovery identities
Botzet et al. (31)	CRP – Exploratory assessment of Augsburg's Step UP Program
Broman et al. (32)	CRP – Community among students without CRP
Broman et al. (5)	CRP – Barriers and facilitators among students without CRP
Broman and Wernekinck (33)	CRP – Resilience among students during COVID-19
Broman et al. (34)	CRP – Recovery capital
Brown and Bohler (35)	CRP – Relapse rate
Brown et al. (36)	CRP – Alumni characteristics
Burns et al. (37)	CRP – Student recovery experiences
Castedo et al. (38)	CRP – Key elements of two effective programs
Castedo de Martell et al. (39)	CRP – Cost effectiveness
Chiang et al. (40)	CRP – Daily craving among students
Clemmons-James (41)	CRP – Historically black colleges and universities
Clemmons-James (42)	CRP – Barriers among black students
Cleveland and Harris (43)	CRP – Conversations with students
Cleveland et al. (44)	CRP – Program characteristics
DePue and Hagedorn (45)	CRP – Facilitating students recovery
Finch and Karakos (46)	CRP – Role of education in recovery
Gerber et al. (47)	CRP – Cost benefit analysis
Giggie et al. (48)	CRP – Mental health comorbidity and recovery among students
Gueci (49)	CRP – Student needs and employee roles
Harris et al. (6)	CRP – Systems based model
Harris et al. (50)	CRP – Program description
Hennessy et al. (51)	CRP – Systematic review
Hennessy et al. (52)	CRP – Emerging adults at public institutions
Hennessy et al. (53)	CRP – Recovery capital
Holtcamp et al. (54)	CRP – Equine interaction during COVID-19
Humberger et al. (55)	CRP – Affirming language, self-compassion, stigma, and recovery capital
Iarussi (56)	CRP – Student recovery experiences
Jurinsky (57)	CRP – Awareness of peers and community
Kimball et al. (58)	CRP – Exploring long term 12-Step experiences of students
Kimball et al. (59)	CRP – Hoping and coping
Knapp et al. (60)	CRP – Exploring daily diary methods and social support
Kollath-Catano et al. (61)	CRP – Challenges and service needs
Laitman and Lederman (62)	CRP – Rutgers continuum of care
Laudet et al. (63)	CRP – Reasons for joining
Laudet et al. (7)	CRP – What we know and what we need to know
Laudet et al. (64)	CRP – Characteristics of students
Monsour et al. (65)	CRP – Challenges of eating disorder recovery programs
Murphy (66)	CRP – Recovery oriented education systems
Murphy et al. (67)	CRP – Acquiring and maintaining spirituality
Nguyen et al. (68)	CRP – First Oxford House Recovery Home
Nichols et al. (69)	CRP – Recovery services needed
Nichols et al. (70)	CRP – Mental and behavioral health conditions
Park et al. (71)	CRP – Participant experiences in Canada
Passman et al. (72)	CRP – Barriers and facilitations to higher education admissions for students
Prior et al. (73)	CRP – Institutional planning
Reed et al. (74)	CRP – Success factors
Romo and Campau (75)	CRP – Managing uncertainty
Smith et al. (76)	CRP – The impact of COVID-19 updated
Smith et al. (77)	CRP – Social support and gender as it relates to relapse risk
Smock et al. (78)	CRP – Social support review of literature
Tabit (79)	CRP – Developing a program from the ground up
Tardif and Stark (80)	CRP – Peer support and the continuum of care
Thompson (81)	CRP – Fostering growth through servant leadership
Vasquez et al. (82)	CRP – Embracing diversity
Vest et al. (83)	CRP – Socio-ecological model
Vest et al. (9)	CRP – PRISMA-guided review of programming
Vest et al. (11)	CRP – Survey of participating students
Vest et al. (10)	CRP – Relationship between criminal legal system and recovery outcomes
Watkins et al. (84)	CRP – Identity transformation
Watts et al. (85)	CRP – Student success
Watts et al. (86)	CRP – Vocational expectations and self-stigmatizing views
Whitney (87)	CRP – Lived experience in three public universities
